# Active removal of waste dye pollutants using Ta_3_N_5_/W_18_O_49_ nanocomposite fibres

**DOI:** 10.1038/s41598-017-04240-4

**Published:** 2017-06-22

**Authors:** Daniel R. Jones, Virginia Gomez, Joseph C. Bear, Bertrand Rome, Francesco Mazzali, James D. McGettrick, Aled R. Lewis, Serena Margadonna, Waheed A. Al-Masry, Charles W. Dunnill

**Affiliations:** 1 Energy Safety Research Institute (ESRI), Swansea University Bay Campus, Swansea, SA1 8EN UK; 2 Materials Chemistry Centre, Department of Chemistry, University College London, 20 Gordon Street, London, WC1H 0AJ UK; 3 College of Engineering, Swansea University Bay Campus, Swansea, SA1 8EN UK; 4 SPECIFIC, Swansea University Bay Campus, Swansea, SA1 8EN UK; 5 Systems and Processing Engineering Centre (SPEC), Swansea University Bay Campus, Swansea, SA1 8EN UK; 6 Department of Chemical Engineering, King Saud University, Riyadh, Saudi Arabia

## Abstract

A scalable solvothermal technique is reported for the synthesis of a photocatalytic composite material consisting of orthorhombic Ta_3_N_5_ nanoparticles and WO_x≤3_ nanowires. Through X-ray diffraction and X-ray photoelectron spectroscopy, the as-grown tungsten(VI) sub-oxide was identified as monoclinic W_18_O_49_. The composite material catalysed the degradation of Rhodamine B at over double the rate of the Ta_3_N_5_ nanoparticles alone under illumination by white light, and continued to exhibit superior catalytic properties following recycling of the catalysts. Moreover, strong molecular adsorption of the dye to the W_18_O_49_ component of the composite resulted in near-complete decolourisation of the solution prior to light exposure. The radical species involved within the photocatalytic mechanisms were also explored through use of scavenger reagents. Our research demonstrates the exciting potential of this novel photocatalyst for the degradation of organic contaminants, and to the authors’ knowledge the material has not been investigated previously. In addition, the simplicity of the synthesis process indicates that the material is a viable candidate for the scale-up and removal of dye pollutants on a wider scale.

## Introduction

With almost 300,000 tonnes of synthetic dye released into the world’s water each year^1^, the environmental damage resulting from such pollutants is a matter of utmost concern. Many of these dyes have been identified as toxic to both aquatic life^2^ and humans^3^, while they also inhibit photosynthetic activity in marine systems due to their strong light absorbance^4^, leading to concerns for global oxygen production. It is reported that more than two billion people across the globe have limited access to clean drinking water^5^, so the need to develop effective, low-cost purification techniques is of increasing importance. Unfortunately, the inherent chemical stability of synthetic dyes typically hinders their degradation by traditional biological treatment methods^6, 7^, while alternative techniques such as coagulation/flocculation^8, 9^ or adsorption on activated carbon^10–12^ are beset by factors such as the production of secondary waste and high material costs. To overcome these shortcomings and to reduce the energy demand of water cleaning relative to conventional energy-intensive techniques, a plethora of novel materials have been developed which act to photocatalyse the oxidation of such recalcitrant waste dyes, and a significant body of research is devoted to increasing the efficiency of these catalysts.

Despite the effectiveness of archetypal TiO_2_ nanopowders in utilising ultraviolet light for dye oxidation^13^, much of the energy from white light remains unused, as the wide band gap of TiO_2_ precludes its absorption of visible light^14^. For this reason, much contemporary research is focussed on the development of narrow band gap photocatalysts such as TaON and Ta_3_N_5_, which possess band gaps of 2.5 eV and 2.1 eV, respectively^15^, as well as WO_3_ and its related Magnéli phase sub-oxides^16^, with corresponding band gaps in the range 2.6–2.8 eV^17–22^. Indeed, in many studies TiO_2_ is used in conjunction with a semiconductor of narrower band gap to yield a composite capable of utilising a much wider range of light wavelengths^20–25^, or alternatively the material may be doped^25–32^ or employed in different phases^31, 33–35^ in an effort to expand the usable portion of the electromagnetic spectrum.

Although both TaON and Ta_3_N_5_ have been shown to degrade organic dyes such as Rhodamine B and methylene blue^36–38^ under exposure to white light, recent work on photocatalysis by Ta_3_N_5_ in the literature suggests that higher degradation rates might be achieved by combining the material with a second, strategically-chosen photocatalyst^39, 40^. By adopting such a two-photon photocatalytic mechanism, the quantum efficiency of the catalyst may be enhanced due to improved separation of electrons and holes within the system^41–52^; one common example of this effect is known as a “Z-scheme”, wherein electron-hole recombination within each material is suppressed due to preferential recombination between the conduction electrons of one component and valence holes of the other^46–52^. In the present investigation, a solvothermal approach^22, 53^ is adapted to grow tungsten(VI) sub-oxide nanostructures on the surface of Ta_3_N_5_ nanoparticles, thereby forming a nanocomposite of the two materials. The photocatalytic properties of the composite are subsequently explored by examining its effect on the oxidation of an aqueous solution of Rhodamine B under illumination by white light.

Tungsten(VI) sub-oxides, most notably W_18_O_49_, have received significant attention in recent years due to their strong absorption of light of visible and even near-infrared wavelengths^54, 55^, high electron mobility^56, 57^ and propensity to form as crystalline nanostructures during solvothermal syntheses^58^. The high conductivity of the material may be partially attributed to the high density of oxygen vacancies, which act as shallow donor states^59^. Moreover, these defects provide adsorption sites for surface species^19, 60, 61^, thereby increasing the rates of surface reactions and improving catalytic activity.

Inspired by similar systems discussed elsewhere^62–64^, the combination of Ta_3_N_5_ and W_18_O_49_ is proposed as an effective system for the oxidation of dye pollutants. As in these previous works, it is demonstrated herein that charge transfer between the two materials successfully enhances the photocatalytic degradation of Rhodamine B relative to the original nanoparticle precursor, both by augmenting the formation of superoxide and hydroxyl radicals and by increasing the rate of direct oxidation of the dye molecules through interaction with valence band holes.

By adopting a solvothermal procedure to grow the W_18_O_49_ heterogeneously from the surface of Ta_3_N_5_ nanoparticles, it is hoped that the present work provides a significant contribution in the quest for photocatalysts that are not only able to degrade dye pollutants in an effective manner, but are also economically viable to produce. Building on previous studies by our group into Janus-like composites^31, 33^, the investigation succeeds in this respect whilst also exploring the synergistic behaviour of two extremely promising photocatalysts which, to the authors’ knowledge, have not been combined for this purpose previously.

## Results

### Nanomaterial characterisation

**Figure 1:**
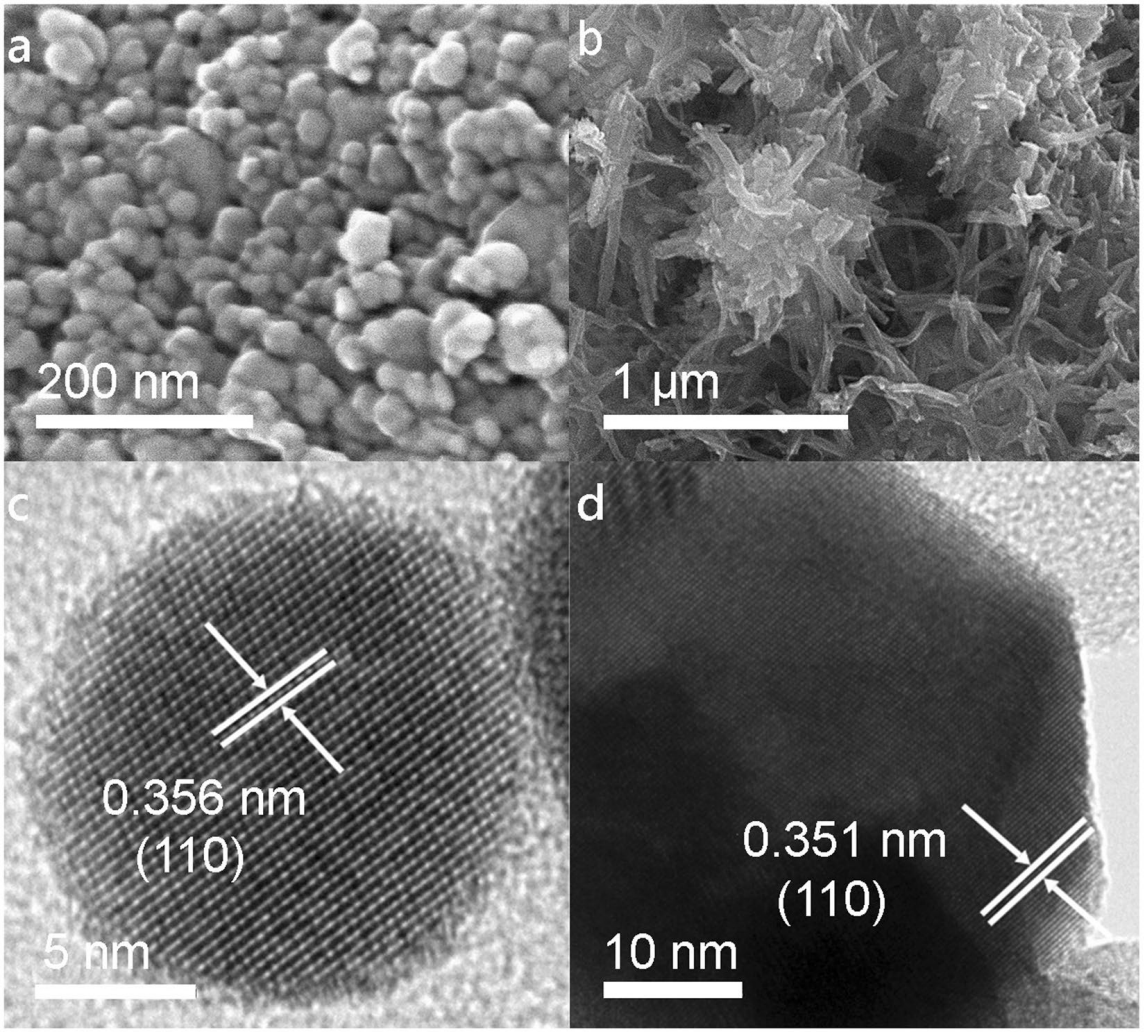
SEM and TEM images showing the Ta_3_N_5_ precursor nanoparticles (**a** and **c**, respectively) and the Ta_3_N_5_/W_18_O_49_ composite (**b** and **d**, respectively). The precursor was shown to contain crystalline nanoparticles of diameter ca. 20–40 nm, while the composite contained both Ta_3_N_5_ nanoparticles and W_18_O_49_ nanowires of mean diameter 32 ± 9 nm.

In order to maximise the surface area of the Ta_3_N_5_/W_18_O_49_ nanocomposite, the thermal decomposition of TaCl_5_ to Ta_3_N_5_ was undertaken inside a eutectic mixture of NaCl and KCl: as this salt mixture melts at a temperature below the annealing temperature of 800 °C, it provided an ionic liquid medium within which the TaCl_5_ could decompose in a controlled manner to form small nanoparticles of Ta_3_N_5_ rather than larger aggregates^65^. As shown by Fig. 1a, which depicts an SEM image of the as-formed Ta_3_N_5_, the nanoparticles typically possessed diameters of ca. 20–40 nm and were approximately spherical in form. Following synthesis of the Ta_3_N_5_/W_18_O_49_ nanocomposite, distinct nanoparticles were more difficult to discern within the structure, possibly due to the presence of a W_18_O_49_ over-layer. By employing TEM to image the Ta_3_N_5_ nanoparticles at atomic resolution, an example of which is displayed in Fig. 1c, the high crystallinity of the nanoparticles became evident; the TEM image shows that the nanoparticles consisted of well-ordered atomic planes, and the measured lattice spacing of 0.356 nm is consistent with the spacing of {110} planes in Ta_3_N_5_, as indexed by JCPDS card number 79–1533.

As typified by the SEM image in Fig. 1b, bundles of W_18_O_49_ nanowires were identified throughout the Ta_3_N_5_/W_18_O_49_ powder, with a mean diameter of 32 ± 9 nm measured over more than forty independent measurements from SEM images. In contrast to similar experiments reported by other groups^22, 53^, the nanowires did not appear to grow uniformly over the Ta_3_N_5_ surface following the formation of a W_18_O_49_ core-shell, but rather there were isolated regions of W_18_O_49_ bundles amongst the Ta_3_N_5_ clusters. It is worth noting, however, that the nanoparticles of Ta_3_N_5_ were significantly smaller than the seeding spheres employed in previous studies, and it is therefore possible that the expected composite morphology was rendered energetically unfavourable as a result of this size disparity. As in Fig. 1c, the TEM image of the composite depicted in Fig. 1d shows a characteristic lattice spacing of 0.351 nm, which again corresponds to the {110} lattice spacing in Ta_3_N_5_. Lattice planes of W_18_O_49_ were much harder to identify, although this may be attributable to the practical difficulty of locating the W_18_O_49_ nanowires due to the abundance of Ta_3_N_5_ nanoparticles.

**Figure 2:**
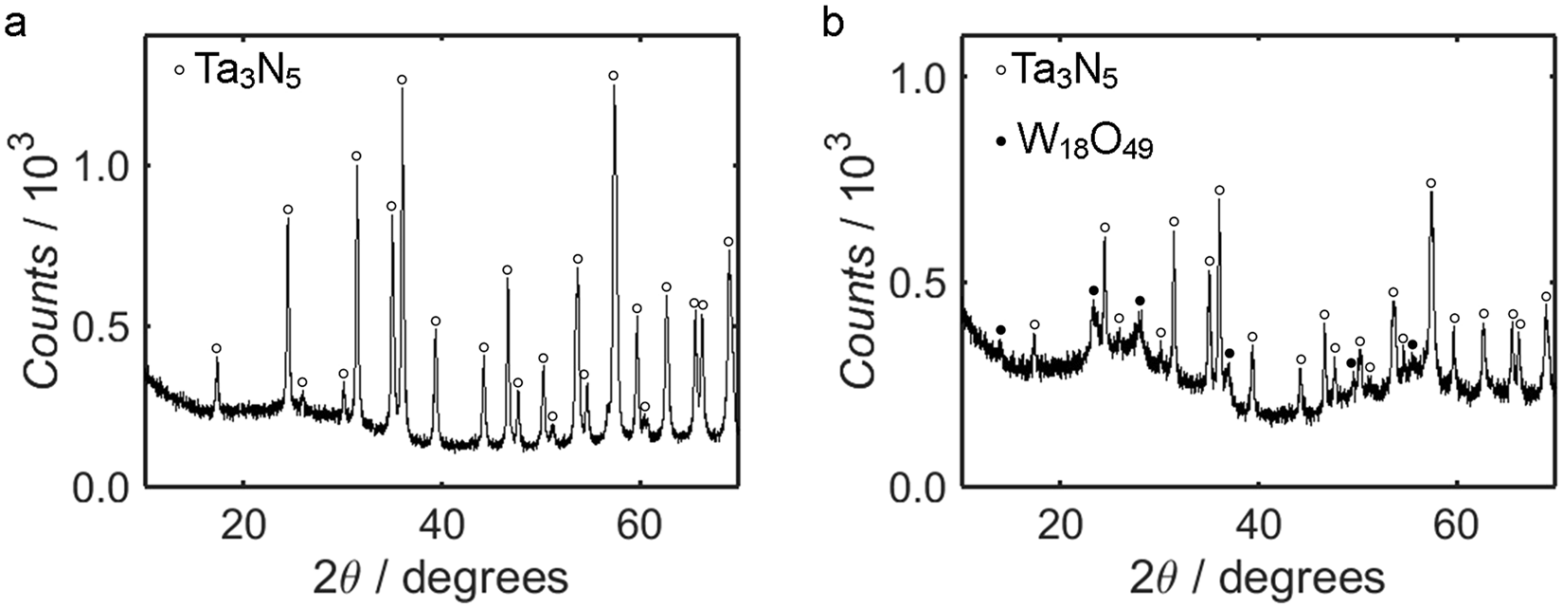
XRD diffractograms of the Ta_3_N_5_ precursor (**a**) and Ta_3_N_5_/W_18_O_49_ nanocomposite (**b**). The peaks corresponding to orthorhombic Ta_3_N_5_ and monoclinic W_18_O_49_ have been indexed using JCPDS card numbers 79–1533 and 71–2450, respectively.

To ascertain the phases of the Ta_3_N_5_ starting precursor and the W_18_O_49_ nanostructures within the Ta_3_N_5_/W_18_O_49_ nanocomposite, an XRD diffractogram was measured for each material. The diffractograms corresponding to Ta_3_N_5_ and Ta_3_N_5_/W_18_O_49_ are displayed in Fig. 2a and b, respectively; it is evident from the plots that while additional peaks exist in the diffractograms of the composite, the majority of the peaks are common to both diffractograms. By comparing the diffractograms to the relevant JCPDS reference cards, the phases of the Ta_3_N_5_ and W_18_O_49_ may be identified as orthorhombic and monoclinic, respectively. Use of the Scherrer equation in conjunction with the Ta_3_N_5_ precursor diffractogram (Fig. 2a) yields an estimate of 22 ± 3 nm for the mean Ta_3_N_5_ nanoparticle diameter, while similar consideration of the Ta_3_N_5_/W_18_O_49_ diffractogram (Fig. 2b) produces a consistent value of 22 ± 4 nm; the shape factor has been allocated a value of 0.9 for these calculations, which is typical for spherical particles^66^, and peaks attributed to the W_18_O_49_ phase have been ignored. The similarity of the two values suggests that the nanoparticle size was not significantly altered during synthesis of the composite.

**Figure 3:**
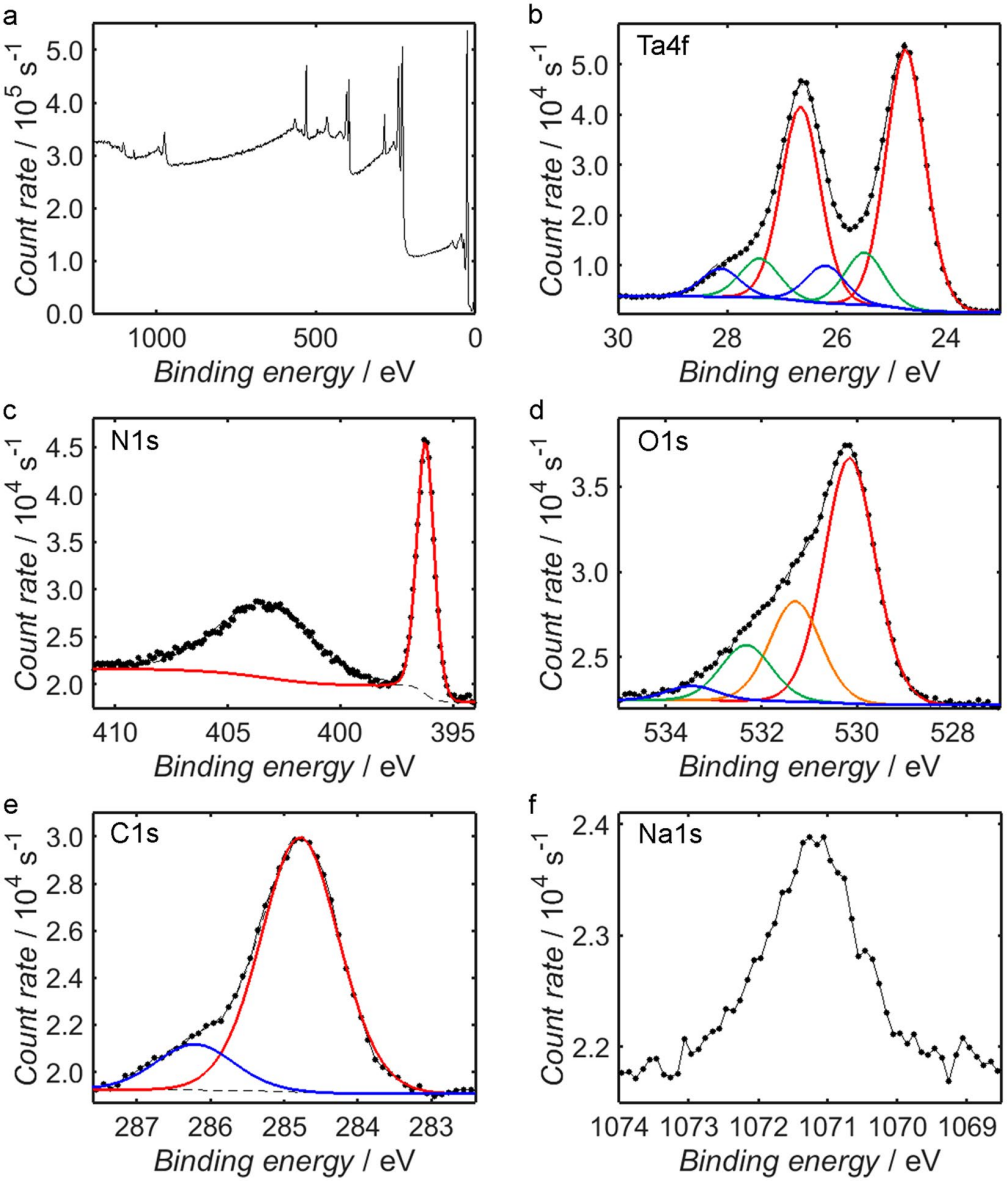
XPS measurements of the Ta_3_N_5_ binding energy spectrum over the range 0–1200 eV (**a**), in addition to higher resolution measurements of the Ta4f (**b**), N1s (**c**), O1s (**d**), C1s (**e**) and Na1s (**f**) peaks. Shirley fits to secondary electron backgrounds are shown as black dashed lines, while fitted peak components are plotted as solid, coloured lines; fitted peaks contained within the same doublet have been assigned the same colour.

The compositions of the Ta_3_N_5_ precursor and the Ta_3_N_5_/W_18_O_49_ nanocomposite were verified using XPS. Figure 3a shows the binding energy spectrum of Ta_3_N_5_ over the range 0–1200 eV, where the energies have been “carbon-corrected” as described in the Methods section. The primary peaks of Ta, N, O, C and Na are displayed in Fig. 3b–f. The presence of a small Na1s peak at 1071 eV, shown in Fig. 3f, suggests that whilst the majority of the NaCl precursor was removed during the annealing and centrifuging steps, some of the Na may have become incorporated into the product, possibly in the form of NaTaO_3_^65^. Such Na-containing species were scarce, however: quantification of the Na1s and Ta4f peaks suggests that the Na to Ta ratio was 1:24. Indeed, all of the peaks in the XRD diffractogram of the precursor in Fig. 2a may be indexed to Ta_3_N_5_, so it is likely that any residual Na compounds were present only on the nanoparticle surface, as opposed to existing in the bulk material. Despite the presence of Na from the eutectic precursor mixture of NaCl and KCl, the absence of a K2p signal suggests that all of the KCl was removed by centrifugation.

The asymmetry of the Ta4f doublet in Fig. 3b indicates that it was contributed to by Ta atoms from several chemical environments; through peak-fitting, it is possible to identify three distinct chemical environments with 4f_7/2_ peaks at binding energies of 24.7 eV, 25.5 eV and 26.2 eV. For each fitting component, it has been ensured that the 4f_5/2_ and 4f_7/2_ peaks have the requisite spin-orbit splitting of 1.9 eV^67^. Comparing to previous literature, one may identify the contribution at 24.7 eV as the Ta_3_N_5_ environment, whereas those at 25.5 eV and 26.2 eV likely correspond to TaON and Ta_2_O_5_, respectively^68–71^. Using these identifications, the proportion of Ta contained within Ta_3_N_5_ is estimated as ca. 74%, with 15% in TaON and 11% in Ta_2_O_5_. As in the case of the Na-containing contaminants, however, the absence of TaON or Ta_2_O_5_ peaks in the XRD diffractogram of the precursor implies that these compounds were present at the surface alone, with the bulk containing negligible quantities of either.

Unlike the Ta4f signal, the N1s peak shown in Fig. 3c is sharp and symmetric and may therefore be fitted adequately by a single component. Unfortunately, there is a slight overlap between this peak and the broad Ta4p_3/2_ peak centred at 403.5 eV, which has a detrimental effect on the precision with which the N1s peak area may be estimated. Recognising this source of error, the area of the N1s peak may be compared to the Ta_3_N_5_ and TaON components of the Ta4f peak in order to evaluate the stoichiometry of the two elements; accounting for the relative proportions of the two nitride species, one anticipates a Ta to N ratio of 0.64, which is fractionally lower than the measured value of 0.70.

**Figure 4:**
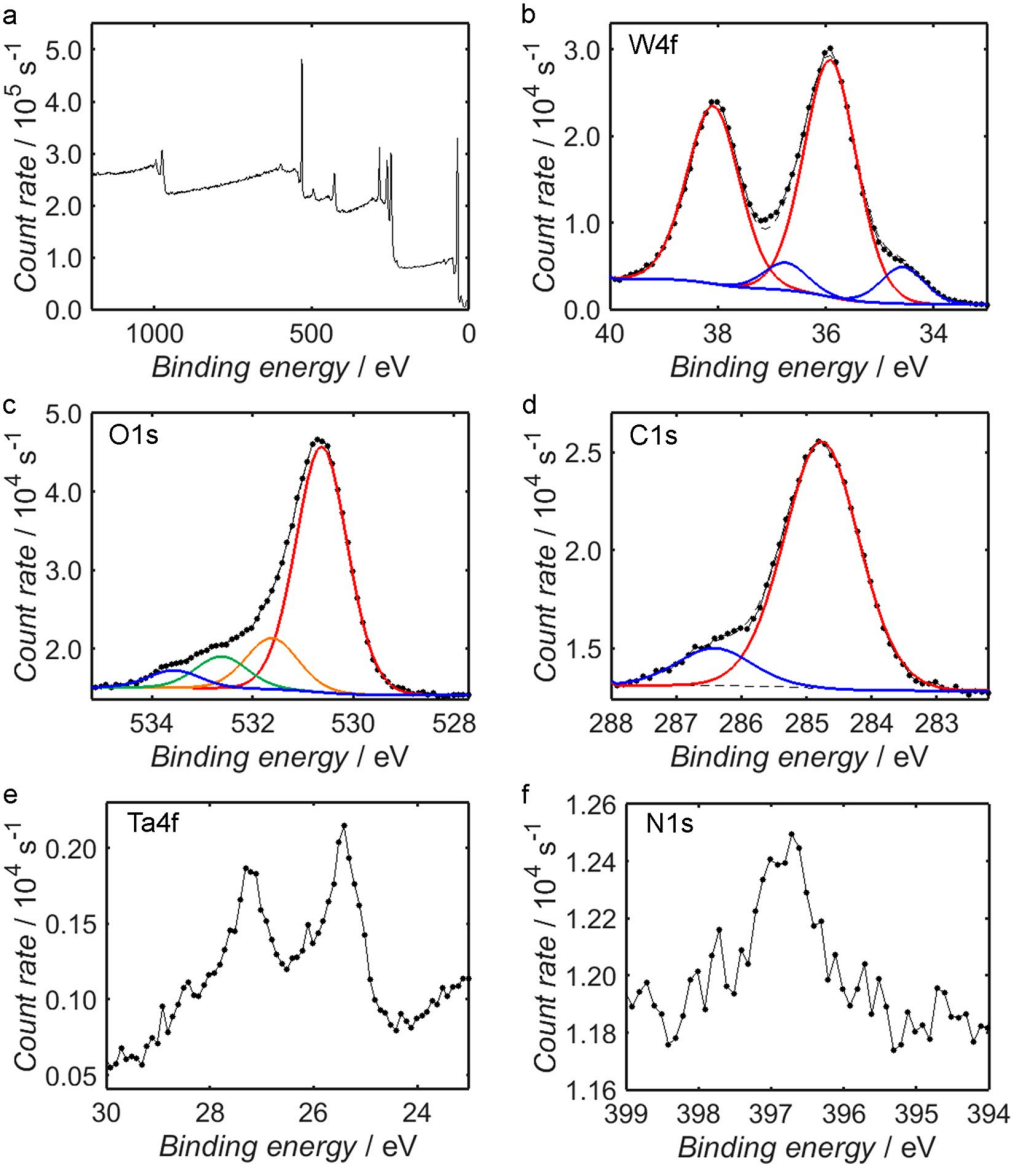
XPS measurements of the Ta_3_N_5_/W_18_O_49_ binding energy spectrum over the range 0–1200 eV (**a**), in addition to higher resolution measurements of the W4f (**b**), O1s (**c**), C1s (**d**), Ta4f (**e**) and N1s (**f**) peaks. Shirley fits to secondary electron backgrounds are shown as black dashed lines, while fitted peak components are plotted as solid, coloured lines; fitted peaks contained within the same doublet have been assigned the same colour.

After forming the Ta_3_N_5_/W_18_O_49_ composite, the presence of nanostructured W_18_O_49_ on the Ta_3_N_5_ surface was expected to diminish the intensity of the Ta4f and N1s signals due to the high surface-sensitivity of the XPS technique. The XPS spectra corresponding to the composite are shown in Fig. 4, and it is clear that the predicted suppression of the Ta4f and N1s peaks, displayed in Fig. 4e and f, respectively, was indeed observed, although the existence of the signals suggests that either the surface coverage by W_18_O_49_ was incomplete or the layer was of sub-10 nm thickness.

To more quantitatively evaluate the composition of the W sub-oxide component of the composite, one must compare the peaks of W and O in a similar manner to those of Ta and N in Fig. 3. In general, sub-oxides of W are mixed-valency compounds containing W^6+^, W^5+^ and W^4+^ species, and it has been demonstrated previously that W_18_O_49_ contains the highest proportion of oxygen vacancies and is the only sub-oxide of W that can be isolated in a chemically-pure form^72^.

By fitting the O1s peak in Fig. 4c with four components, it is possible to identify the relative proportions of the different oxygen environments; in accordance with previous work, one may attribute the component at lowest binding energy to oxygen contained within the sub-oxide lattice, while the adjacent component is thought to correspond to surface hydroxyl groups^73, 74^. The final two contributions to the O1s peak are typically assigned to adsorbed water and adventitious organic compounds^54, 73–76^. It should be noted that the O1s peak may also contain contributions from TaON and Ta_2_O_5_ species at the Ta_3_N_5_ surface, although these components are likely insignificant due to the near-complete coverage by W_18_O_49_.

The different oxidation states of W are represented by independent doublets within the W4f signal, as depicted in Fig. 4b; for each component, the separation between the 4f_5/2_ and 4f_7/2_ spin states has been assigned the characteristic value of 2.17 eV^77^. According to previous literature, the 4f_7/2_ peak attributed to the W^6+^ ion is centred at a binding energy of between 35.4 eV and 36.7 eV, while those of W^5+^ and W^4+^ appear in the ranges 34.0–35.0 eV and 32.2–34.1 eV, respectively^54, 55, 60, 73–76, 78–81^. In the present case, it is demonstrated in Fig. 4b that the W4f signal may be fitted adequately by two sets of doublets, with the two 4f_7/2_ peaks appearing at binding energies of 35.9 eV and 34.6 eV; from the previous identifications, these peaks may be attributed to W^6+^ and W^5+^ ions, respectively. By comparing the total area of the W4f signal to the combined area of the sub-oxide components within the O1s peak, accounting for both oxygen contained within the bulk lattice and surface oxygen bound to atoms of hydrogen to form hydroxyl groups, one obtains an O to W atomic ratio of 2.69, which is in close agreement with the anticipated value of 2.72.

**Figure 5:**
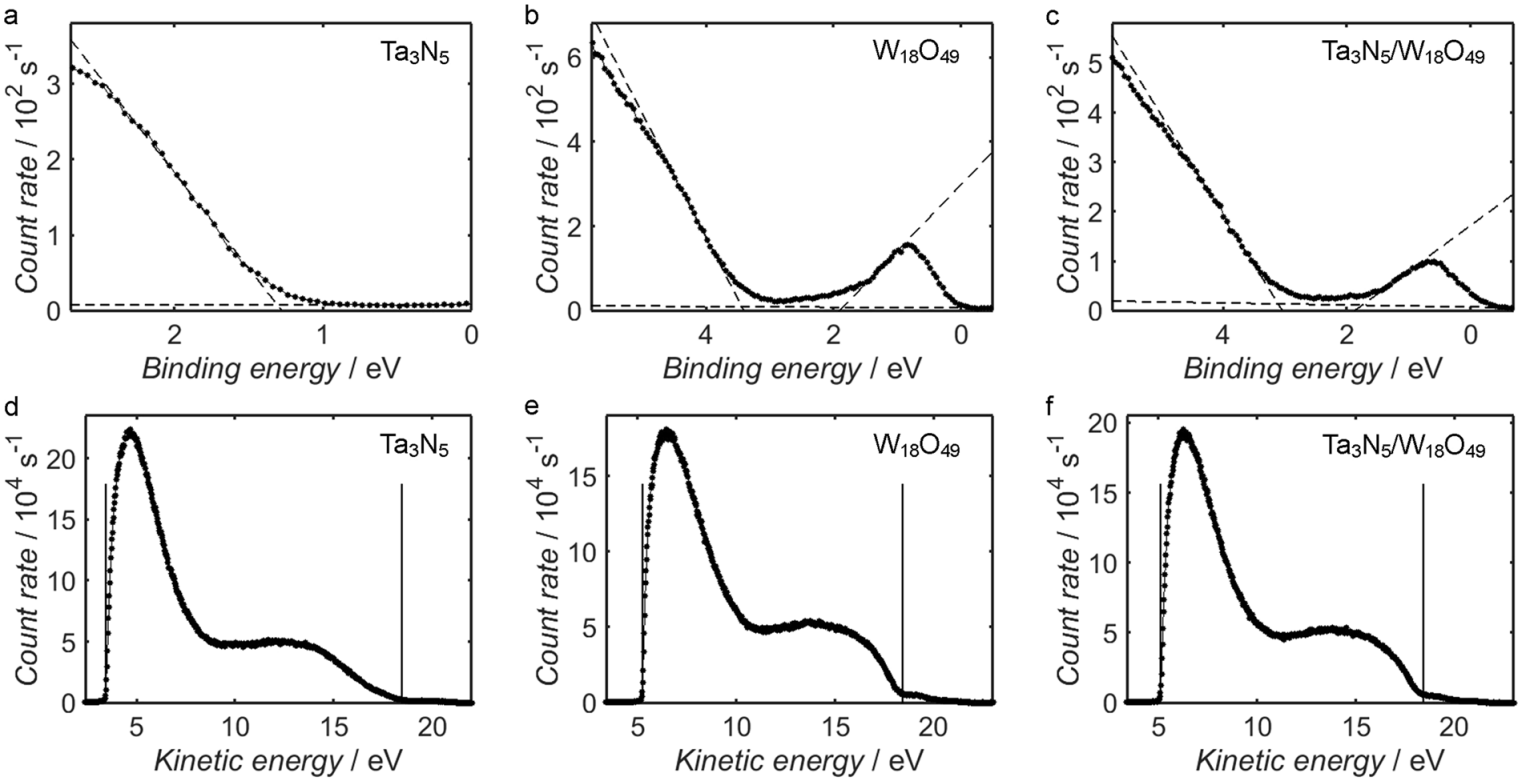
XPS measurements of the valence band edge of Ta_3_N_5_ (**a**), W_18_O_49_ (**b**) and Ta_3_N_5_/W_18_O_49_ (**c**), and UPS measurements corresponding to the same materials (**d**–**f**). Linear fits to the valence band edge and the baseline are depicted as dashed lines in (**a**–**c**), the intersection of which may be interpreted as the energy of the valence band maximum, *E*_V_, relative to the Fermi level, *E*_F_. In (**b**,**c**), a similar procedure has been carried out to calculate the position of the conduction band minimum, *E*_C_. The estimated positions of *E*_V_ and the secondary electron onset, *E*_SEO_, from UPS are shown in plots (**d**–**f**) as solid vertical lines.

In addition to verifying the elemental compositions of the catalysts, XPS is also useful for determining the band structures of the two materials. In Fig. 5, measurements of the XPS spectrum of each sample are shown for binding energies close to zero, which coincides with the Fermi level, *E*_F_, of the system. In each case, the intensity of the spectrum decreased sharply upon approaching the Fermi level; this feature of the spectrum corresponds to the edge of the valence band, and the position of the valence band maximum, *E*_V_, relative to *E*_F_ may be estimated from the intersection of a line-of-best-fit through the points corresponding to the valence band edge and a second linear fit through the baseline of the scan^82–84^. From the linear fits depicted as dashed lines in Fig. 5a–c, one obtains estimated *E*_F_-*E*_V_ values of 1.3 eV for Ta_3_N_5_, 3.4 eV for W_18_O_49_ and 3.1 eV for Ta_3_N_5_/W_18_O_49_. The similarity between the valence band scans of W_18_O_49_ and the composite provides further evidence that W_18_O_49_ existed as a shell on the Ta_3_N_5_ surface.

In the cases of W_18_O_49_ and the composite, occupied states were measured just below the Fermi level in the XPS valence band measurements, suggesting that the conduction band of W_18_O_49_ extended below *E*_F_; this property is characteristic of a degenerate semiconductor, wherein the concentration of donor states is sufficiently high to fully-occupy states close to the conduction band edge. Such behaviour has been observed previously in XPS spectra of W sub-oxides and attributed to the high concentration of oxygen vacancies, and the signal is therefore absent in similar measurements acquired from pristine WO_3_ films^85–87^. By applying a linear fit to the conduction band edge in a similar manner to the valence band, the conduction band minimum of W_18_O_49_, *E*_C_(W_18_O_49_), is estimated at ca. 1.9 eV below *E*_F_, while a value of 1.8 eV is obtained for Ta_3_N_5_/W_18_O_49_; these energy values are commonly known as the Moss-Burstein shift^88^. The difference between *E*_V_ and *E*_C_ yields an estimated electronic band gap of 1.6 eV for W_18_O_49_ and 1.4 eV for Ta_3_N_5_/W_18_O_49_, and the measured positions of the bands are consistent with those predicted from *ab initio* DFT calculations reported in the literature^16, 89^. It should be noted that the optical band gap of the material is equal to the difference between *E*_V_ and *E*_F_, as photoexcited valence electrons cannot be promoted to occupied states at the base of the conduction band.

Since the XPS measurements in Fig. 5a–c have been carbon-corrected to account for any electric potential at the sample surface, *E*_F_ is located at ca. 0 eV in each case and it is therefore possible to calculate the difference between *E*_V_ estimates of two different samples. One cannot, however, deduce the ionisation potentials, *E*_IP_ , of the three materials from these measurements, as they provide no information about the energy of the vacuum level, *E*_VAC_. To estimate the ionisation potential, it is therefore necessary to employ UPS: the energy difference between *E*_V_ and the energy of the secondary electron onset, *E*_SEO_, corresponds to the difference between the energy of incident photons and the ionisation potential of the material. The UPS measurements from the three samples are shown in Fig. 5d–f, along with the positions of *E*_V_ and *E*_SEO_ estimated from linear fits to the valence band edge and the edge of the secondary electron peak, respectively; these fits are shown in Supplementary Fig. S1, and *E*_V_ and *E*_SEO_ have been estimated from the intersection between each linear fit and a further fits through the baseline. From the *E*_V_ and *E*_SEO_ positions shown, one obtains an *E*_IP_ value of 6.2 eV for Ta_3_N_5_, 8.0 eV for W_18_O_49_ and 7.9 eV for Ta_3_N_5_/W_18_O_49_, which are consistent with values reported in the literature^19, 20, 71, 90^; it is also worth noting that the differences between these three *E*_IP_ values are in close agreement with the differences between the *E*_V_ estimates from Fig. 5a–c.

In addition to the ionisation potential, it is possible to use UPS measurements to calculate the work function, *ϕ*, of a metallic or semiconducting material. This is achieved by first approximating the Fermi energy of the system, which for suitably conducting samples may be assumed equal to the Fermi energy of the spectrometer, as the centre-point of the Fermi edge in UPS measurements of the metallic sample holder itself (as shown in Supplementary Fig. S2). One may then estimate *ϕ* by calculating the difference between the photon energy and *E*_SEO_-*E*_F_. Due to the semi-metallic nature of W_18_O_49_ and the composite, the assumption of electronic equilibrium between spectrometer and sample is appropriate, and *ϕ* estimates of 4.8 eV and 4.7 eV are obtained for the two materials, respectively. Subtracting these values from the previous estimates of *E*_IP_ yields an *E*_F_-*E*_V_ value of 3.2 eV for both materials, which is consistent with the corresponding estimates from Fig. 5b and c. In the case of Ta_3_N_5_, however, the *ϕ* estimate of 3.0 eV from Fig. 5d is considerably smaller than the value of 4.9 eV expected from *E*_IP_(Ta_3_N_5_) and the *E*_F_-*E*_V_(Ta_3_N_5_) estimate from Fig. 5a; this disparity is likely due to the electrically insulating nature of Ta_3_N_5_, with the resulting surface charging leading to a difference in the Fermi level between sample and spectrometer.

**Figure 6:**
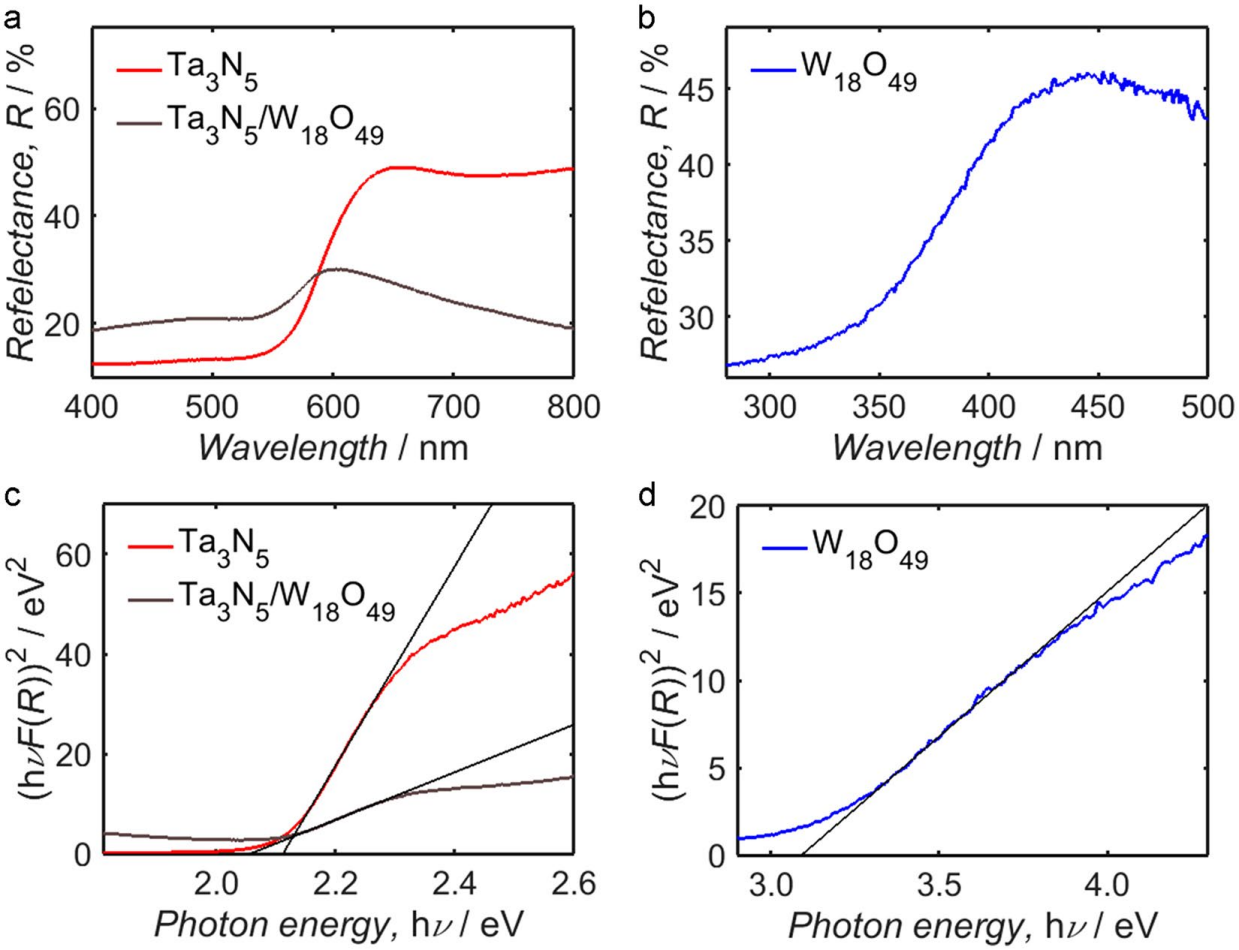
Diffuse reflectance spectra of Ta_3_N_5_ and Ta_3_N_5_/W_18_O_49_ (**a**), as well as W_18_O_49_ (**b**), with corresponding Tauc plots in (**c**,**d**), respectively. By plotting a line-of-best-fit through the point of maximum gradient within each Tauc plot, an estimate of the optical band gap may be obtained; values of ca. 2.1 eV are estimated for both Ta_3_N_5_ and Ta_3_N_5_/W_18_O_49_, whereas the measurements from W_18_O_49_ yield a value of ca. 3.1 eV.

Further information regarding the electronic band structure of a material is provided by UV-Vis diffuse reflectance spectroscopy. By constructing Tauc plots from the UV-Vis diffuse reflectance measurements depicted in Fig. 6a and b, as shown in Fig. 6c and d, it is possible to estimate the optical band gaps of Ta_3_N_5_, W_18_O_49_ and the Ta_3_N_5_/W_18_O_49_ composite. In this way, a band gap estimate of 2.11 ± 0.03 eV is obtained from the Tauc plot of the Ta_3_N_5_ precursor, which is consistent with the accepted value for Ta_3_N_5_, while a similar value of 2.06 ± 0.03 eV is yielded by measurements from the Ta_3_N_5_/W_18_O_49_ composite. The band gap of W_18_O_49_, estimated from the Tauc plot in Fig. 6d, has a much higher value of 3.09 ± 0.05 eV, which is comparable to the values obtained from the XPS and UPS measurements in Fig. 5b and e, respectively; it should be recognised that it is not strictly appropriate to compare the band gap estimates from the different experimental techniques, as the Fermi level of the material likely varied between instruments. In the case of Ta_3_N_5_/W_18_O_49_, no step in the diffuse reflectance was measured at wavelengths lower than 400 nm, suggesting that W_18_O_49_ accounted for a much lower proportion of the composite than the Ta_3_N_5_ component.

### Composite band structure

**Figure 7:**
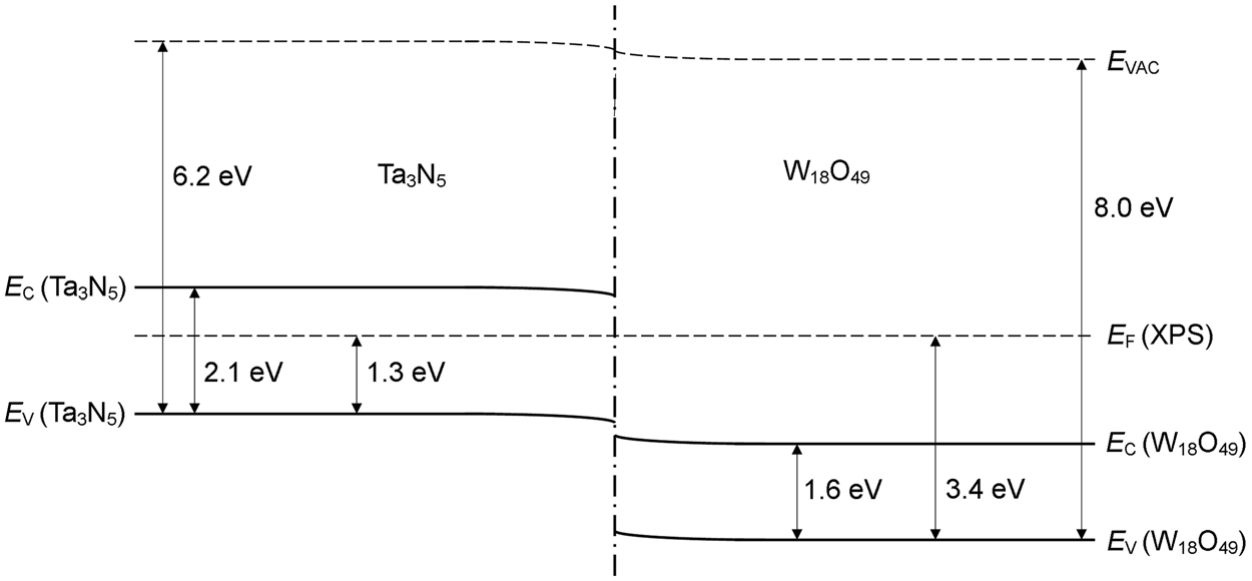
Proposed band structure of Ta_3_N_5_/W_18_O_49_, constructed using estimates of the optical band gaps of Ta_3_N_5_ and W_18_O_49_ from Fig. 6, as well as *E*_IP_ estimates from Fig. 5d and e, and the estimated electronic band gap of W_18_O_49_ from Fig. 5b. The position of *E*_F_ shown corresponds to the Fermi level estimated from the XPS valence band measurements in Fig. 5a and b; however, the true Fermi level of the isolated composite is not known, so the depicted position serves solely to illustrate the semi-metallic nature of the W_18_O_49_ component.

By combining the deductions from Figs 5 and 6, one may visualise the relative positions of the energy bands in Ta_3_N_5_/W_18_O_49_; as illustrated in Fig. 7, the conduction and valence band edges of W_18_O_49_ were located at a significantly higher redox potential than the edges of the two bands in Ta_3_N_5_. The annotated energy values have been compiled using the optical band gap estimates from Fig. 6, estimated *E*_IP_ values from Fig. 5d and e and the electronic band gap of W_18_O_49_ estimated from Fig. 5b. Although the value of *E*_F_ in the isolated composite powder was likely different to the *E*_F_ value that existed during XPS and UPS measurements, the *E*_F_-*E*_V_ estimates from Fig. 5a and b have been used to provide a semi-qualitative *E*_F_ position for the diagram and thereby illustrate the semi-metallic behaviour of W_18_O_49_; despite the variability of the *E*_F_ position, however, it is worth recalling the similarity between the *E*_F_ estimate obtained from the UV-Vis diffuse reflectance measurements of W_18_O_49_ and XPS valence band measurements of the same material.

The band alignments in Fig. 7 suggest the possibility of a Z-scheme of electron transfer between Ta_3_N_5_ and W_18_O_49_: following photo-excitation of valence electrons, electron-hole recombination within each material may be suppressed due to preferential recombination of W_18_O_49_ conduction electrons with holes from the valence band of Ta_3_N_5_. The resulting increase in the density of conduction electrons in Ta_3_N_5_ would be expected to enhance the rate of reductive reaction processes at the Ta_3_N_5_ surface, whereas the increased hole concentration in W_18_O_49_ would correspondingly promote the oxidation of surface species.

### White light photocatalysis and cyclability

The application of the Ta_3_N_5_/W_18_O_49_ composite as a photocatalyst for Rhodamine B oxidation was tested alongside the Ta_3_N_5_ precursor and W_18_O_49_ control powder. Prior to illumination of the catalyst suspensions in Rhodamine B solution (0.02 mM), the absorption of the dye was measured after one hour of stirring in darkness; it was discovered that the Ta_3_N_5_/W_18_O_49_ composite decreased the peak absorption intensity by 91% relative to a control solution containing no catalyst, whereas the Ta_3_N_5_ precursor decreased the peak intensity by just 4%. In the W_18_O_49_ control suspension, the absorption peak of the dye reduced by 97% relative to the solution containing Rhodamine B alone. These measurements suggest that the dye adsorbed strongly to the W_18_O_49_ shell of the Ta_3_N_5_/W_18_O_49_ composite, likely due to surface oxygen vacancies in W_18_O_49_ serving as favourable sites for molecular adsorption^80, 91, 92^.

**Figure 8:**
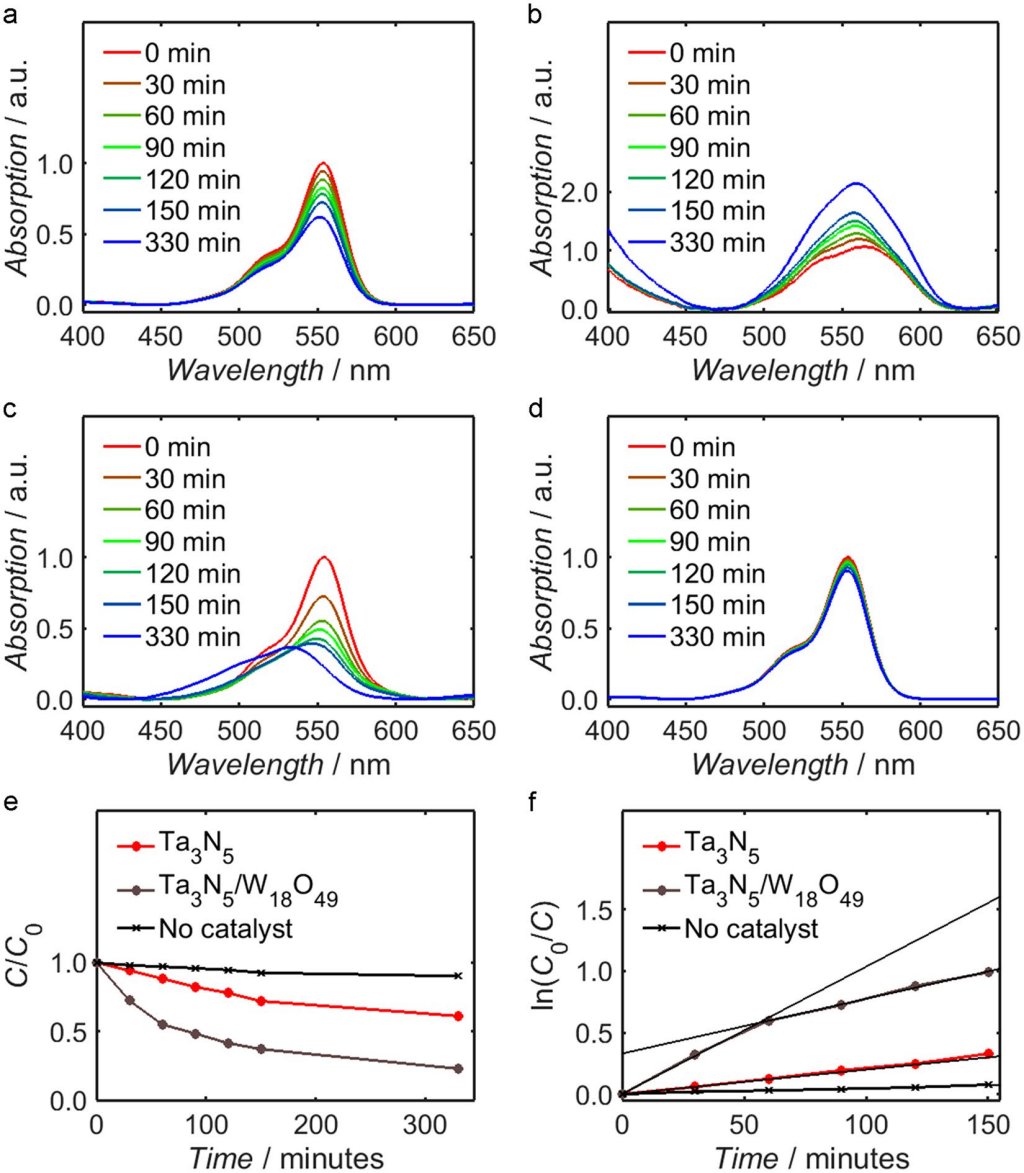
UV-Vis absorption spectra of supernatant extracted from white light illuminated suspensions of Ta_3_N_5_ (**a**), W_18_O_49_ (**b**) and Ta_3_N_5_/W_18_O_49_ (**c**) in Rhodamine B solution (0.02 mM), as well as a control solution containing no catalyst (**d**). For Ta_3_N_5_, Ta_3_N_5_/W_18_O_49_ and the control solution, the progression of absorption at 554 nm, *C*, is plotted as a function of white light illumination time in (**e**), normalised with respect to the value of *C* prior to illumination, *C*_0_. The ratio *C*_0_/*C* is also plotted logarithmically for these samples in (**f**). The plots show that while W_18_O_49_ had no observable catalytic effect on dye oxidation, Ta_3_N_5_/W_18_O_49_ photocatalysed the reaction at over double the rate of Ta_3_N_5_ on its own. The composite also yielded the highest rate of N-deethylation, as shown by the pronounced hypsochromic shift of the absorption peak in (**c**).

During stepwise illumination of the suspensions, both Ta_3_N_5_ and Ta_3_N_5_/W_18_O_49_ had a catalytic effect on the oxidation of Rhodamine B: as shown by Fig. 8a and c, in each case the UV-Vis absorption spectrum of the dye solution diminished in intensity between successive steps more rapidly than in the control solution, for which the corresponding measurements are displayed in Fig. 8d. Conversely, Fig. 8b indicates that W_18_O_49_ alone had negligible catalytic effect on dye oxidation; indeed, the intensity of the absorption peak actually increased with successive illumination steps, possibly due to gradual desorption of adsorbed dye from the catalyst surface. It should be noted that in all four spectra the absorption has been normalised with respect to the absorption at 554 nm of the same sample prior to illumination but after stirring in darkness, denoted *C*_0_. The unusual shape of the spectra in Fig. 8b, corresponding to the W_18_O_49_ suspension, may be attributed to the low concentration of dye molecules in solution following adsorption on the catalyst: the absolute absorption intensity was consequently significantly lower than in the other samples, and the form of the spectrum was therefore likely altered by background noise within the signal.

The difference in the rate of photolysis between the two active catalysts, Ta_3_N_5_ and Ta_3_N_5_/W_18_O_49_, is illustrated more explicitly by Fig. 8e, which depicts the variation of absorption at 554 nm, *C*, normalised with respect to *C*_0_, as a function of illumination time; after 150 minutes of white light exposure there was a 63% decrease in the Rhodamine B peak intensity in the presence of Ta_3_N_5_/W_18_O_49_, compared to just 28% for the suspension containing Ta_3_N_5_ only. Due to the disparate illumination setups used by different research groups, it is unfortunately difficult to compare the measured rates of decolourisation to those attributed to other catalysts in the literature. It is informative, however to consider a previous study by our group in which the same light box was used, and it is evident that the Ta_3_N_5_/W_18_O_49_ nanocomposite is indeed competitive with the anatase/rutile TiO_2_ composites explored therein: employing 1 mg mL^−1^ of suspended catalyst in 0.01 mM Rhodamine B solution, the anatase/rutile TiO_2_ catalyst yielded a 70% decrease in the Rhodamine B peak intensity after 150 minutes, which is only fractionally higher than the decolourisation achieved in the present study by 0.5 mg mL^−1^ of Ta_3_N_5_/W_18_O_49_ in 0.02 mM Rhodamine B solution^31^.

By further displaying the natural logarithm of *C*_0_/*C* as a function of time, Fig. 8f allows quantitative estimation of the oxidation rate in each suspension. Although the intensity at 554 nm decreased linearly as a function of time in the case of Ta_3_N_5_, the corresponding plot for Ta_3_N_5_/W_18_O_49_ is comprised of two distinct regimes with different rate constants. Through linear fitting of these two regions of the plot, one obtains an estimated decolourisation rate constant of 1.0 × 10^−2^ min^−1^ for times of up to one hour and a value of 4.5 × 10^−3^ min^−1^ for longer times; it is probable that the transiently high rate of decolourisation at short times may be attributed to adsorption of the dye at the catalyst surface. Notably, the rate constant for longer durations of illumination is more than double the corresponding estimate of 2.0 × 10^−3^ min^−1^ for the Ta_3_N_5_ precursor; this result, in conjunction with the observed inactivity of the W_18_O_49_ control sample, suggests that synergistic effects between Ta_3_N_5_ and W_18_O_49_ within the composite acted to enhance the efficiency of photocatalysis relative to Ta_3_N_5_ alone. As mentioned previously, comparison of the measured rate constants to previous works is problematic due to the different apparatus used, but it is nevertheless instructive to note that similar values of 1.0 × 10^−3^–4.0 × 10^−2^ min^−1^ are typically reported for Rhodamine B degradation under visible light irradiation^93–100^.

Another important factor affecting the commercial viability of a photocatalyst is its cyclability. To this end, the three sample catalysts were recovered from their respective dye solutions through repeated centrifugation into deionised water, before the entire sequence of illumination cycles and UV-Vis absorption experiments was repeated using fresh solutions of Rhodamine B; the results from this sequence of experiments are plotted in Supplementary Fig. S3a–d, wherein the UV-Vis absorption spectra following different periods of illumination have again been normalised with respect to *C*_0_ and overlaid, while Supplementary Fig. S3e shows the time variations of the normalised Rhodamine B absorption at wavelength 554 nm. To determine rate constant estimates for dye oxidation in the presence of each catalyst, *C*_0_/*C* has also been plotted logarithmically as a function of illumination time in Supplementary Fig. S3f.

During the initial hour of stirring in darkness, the quantity of dye adsorbed by recycled Ta_3_N_5_/W_18_O_49_ and W_18_O_49_ was significantly lower than for the as-synthesised powders; however, relative to the solution containing no catalyst, the two materials still decreased the peak absorption intensity of the dye by 24% and 95%, respectively, compared to a smaller decrease of 11% in the case of recycled Ta_3_N_5_. Once again, it is clear from Supplementary Fig. S3e,f that the Ta_3_N_5_/W_18_O_49_ nanocomposite oxidised the dye at a superior rate to the Ta_3_N_5_ precursor alone, while W_18_O_49_ continued to have no apparent catalytic effect on the rate of dye photolysis. Moreover, Fig. S3f indicates that both Ta_3_N_5_ and Ta_3_N_5_/W_18_O_49_ again catalysed the decolourisation of Rhodamine B solution at an approximately exponential rate, albeit more slowly than during the first illumination sequence. In the case of Ta_3_N_5_, the absorption peak was found to decrease at a transiently low rate during the first hour of illumination, so the corresponding linear fit in Supplementary Fig. S3f has been applied only to points associated with illumination times of one hour or more. As before, the Ta_3_N_5_/W_18_O_49_ composite catalysed the rate of dye oxidation at approximately double the rate of Ta_3_N_5_ on its own: from the linear fits depicted in Supplementary Fig. S3f, one obtains estimated rate constants of 3.8 × 10^−3^ min^−1^ and 2.2 × 10^−3^ min^−1^ for Ta_3_N_5_/W_18_O_49_ and Ta_3_N_5_, respectively.

From the variation of the Rhodamine B absorption spectra during each set of illumination sequences, both before and after recycling of the catalysts, it is possible to make qualitative deductions regarding photocatalytic mechanisms within the Ta_3_N_5_/W_18_O_49_ system. For instance, both the as-synthesised and recycled Ta_3_N_5_/W_18_O_49_ composite led to a pronounced “hypsochromic” shift of the absorption maximum after prolonged illumination. For the as-synthesised material (Fig. 8c), the peak was found to shift from a wavelength of 554 nm to 532 nm after 120 minutes of illumination, followed by the emergence of a distinct absorption component at ca. 500 nm after 330 minutes of white light exposure. Supplementary Fig. S3c shows that recycled Ta_3_N_5_/W_18_O_49_ resulted in a much slower rate of hypsochromic shifting, with negligible variation in peak position after 150 minutes of illumination; the absorption spectrum after 1110 minutes, however, exhibits the same shift of the absorption peak to 500 nm. This behaviour is typically attributed to the formation of photoactive intermediates following stepwise N-deethylation of the Rhodamine B molecules; more specifically, four ethyl groups are removed sequentially from each molecule in the form of acetaldehyde, thereby generating three successive intermediates followed by Rhodamine 110 as the final product^101–106^. The position of the 500 nm absorption component after extensive illumination is consistent with the reported absorption maximum of Rhodamine 110.

In contrast to the composite, the as-synthesised Ta_3_N_5_ precursor (Fig. 8a) did not produce a significant shift in the wavelength of peak dye absorption after 330 minutes of illumination, although a shift to 535 nm occurred after 1110 minutes in the presence of recycled Ta_3_N_5_ (Supplementary Fig. S3a); it is apparent, therefore, that while N-deethylation of Rhodamine B was possible at the Ta_3_N_5_ surface, the catalysis mechanism predominantly involved cleavage of the Rhodamine B chromophore to non-photoactive products. Similarly, the W_18_O_49_ control powder yielded negligible hypsochromic shift over the timeframe of the first set of illumination steps (Fig. 8b), although a small, but significant, shift was produced following stepwise illumination of the dye in the presence of recycled W_18_O_49_ for 1110 minutes (Supplementary Fig. S3b). Combined with the results from the Ta_3_N_5_/W_18_O_49_ measurements, these observations suggest that the reactive species responsible for N-deethylation of the dye were generated at both Ta_3_N_5_ and W_18_O_49_ surfaces, but the rate of their generation was enhanced by electron transfer between Ta_3_N_5_ and W_18_O_49_ within the composite.

To further elucidate the species involved in the photocatalytic mechanisms and their specific roles within them, stepwise illumination of Rhodamine B solution was performed with suspended Ta_3_N_5_ or Ta_3_N_5_/W_18_O_49_ and the addition of either *tert*-butanol or *p*-benzoquinone as scavengers of hydroxyl and superoxide radicals, respectively^64, 107–110^. Both of these species may form due to redox processes at a catalyst surface, subject to the energies of the conduction and valence bands of the material relative to the redox potentials of the radical formation reactions; it is worth noting, for instance, that the oxidation of water molecules to hydroxyl radicals and protons has an associated redox potential of 6.81 eV relative to vacuum^111, 112^, so could not have occurred at the Ta_3_N_5_ surface as the valence band edge was located at higher energy, as shown by Fig. 7. By contrast, the reduction of dissolved molecular oxygen by Ta_3_N_5_ to form superoxide radicals was thermodynamically feasible: following photo-excitation, electrons at the conduction band edge, which Fig. 7 suggests had an energy of ca. 4.1 eV below *E*_VAC_, could partake in the formation of these radicals, as the reaction possesses a higher redox potential of 4.32 eV relative to vacuum^112^. Similar reasoning suggests that W_18_O_49_ could successfully generate hydroxyl radicals, while it may have also produced superoxide radicals by first coordinating with hydroxyl radicals to form sites at which the reduction of molecular oxygen was energetically favourable^113^. Both Ta_3_N_5_ and W_18_O_49_ were ostensibly able to photocatalyse chromophore cleavage through oxidation of Rhodamine B molecules in either their photo-excited or relaxed state^110, 114^. It is worth noting that if, as proposed previously, electron-hole recombination was suppressed by a Z-scheme of electron transfer in the composite, the resulting enhancement of hole concentration in W_18_O_49_ possibly acted to augment production of hydroxyl radicals through water oxidation, while the corresponding increase in the number of conduction electrons in Ta_3_N_5_ may similarly have increased the formation rate of superoxide radicals from the reduction of dissolved oxygen.

The effects of stepwise illumination on the Ta_3_N_5_ and Ta_3_N_5_/W_18_O_49_ suspensions in the presence of scavengers are shown in Supplementary Figs S4 and S5, respectively. Despite the expected hypsochromic shift occurring within the Ta_3_N_5_/W_18_O_49_ suspensions containing either no scavenger reagent or *tert*-butanol (Supplementary Fig. S5a,c, respectively), the phenomenon was completely suppressed by *p*-benzoquinone (Supplementary Fig. S5b); it is probable, therefore, that the N-deethylation of Rhodamine B was dependent on the presence of superoxide radicals within the system. Over the 150 minute timeframe of the experiment, negligible hypsochromic shifting was observed in suspensions containing the Ta_3_N_5_ precursor catalyst, indicative of a much lower formation rate of superoxide radicals.

To analyse the influence of each scavenger reagent on the rate of chromophore cleavage, Supplementary Fig. S6 plots the absorption intensity of the dye at 554 nm as a function of illumination time for both the Ta_3_N_5_ suspensions (Supplementary Fig. S6a) and those containing Ta_3_N_5_/W_18_O_49_ (Supplementary Fig. S6b). In contrast to studies elsewhere in the literature, in which *tert*-butanol and *p*-benzoquinone acted to diminish the rate of dye oxidation^64, 107–110^, no such decrease was witnessed in the present experiment; indeed, Supplementary Fig. S6a shows that *p*-benzoquinone actually increased the rate of degradation in the Ta_3_N_5_ suspension, whereas both reagents enhanced the decolourisation rate by a similar factor when employed alongside Ta_3_N_5_/W_18_O_49_.

It is unclear how the removal of superoxide radicals from the Ta_3_N_5_ system served to enhance cleavage of the Rhodamine B chromophore, but one possibility is that their continuous removal resulted in a lower concentration of conduction band electrons, thereby decreasing electron-hole recombination and yielding an increased density of valence holes for the oxidation of Rhodamine B molecules. The use of *tert*-butanol, a scavenger of hydroxyl radicals, had no significant influence on the rate of Rhodamine B decolourisation in this system, which is consistent with the predicted infeasibility of water oxidation by Ta_3_N_5_. By contrast, the pronounced effects of *tert*-butanol and *p*-benzoquinone on decolourisation rate in Ta_3_N_5_/W_18_O_49_ suspension suggest that not only were hydroxyl and superoxide radicals readily formed within this system, but that they were both active species in the cleavage mechanism of the Rhodamine B chromophore. The poor catalytic activity of W_18_O_49_ on its own is therefore suggestive of an inability to generate either radical species at an appreciable rate in the absence of Ta_3_N_5_, so the presence of these radicals in the composite system may be directly attributed to the influence of electronic transfer between the two materials.

## Conclusions

By employing a facile and potentially scalable solvothermal procedure, a highly effective Ta_3_N_5_/W_18_O_49_ nanocomposite has been synthesised for the photodegradation of dye pollutants in wastewater. In comparison to the Ta_3_N_5_ precursor alone, the composite material achieved over double the rate of Rhodamine B oxidation under white light illumination, while also removing over 90% of the dissolved dye through molecular adsorption, in a manner analogous to activated carbon used commercially. The cyclability of the composite was also demonstrated: following cleaning of the materials in deionised water, suspended Ta_3_N_5_/W_18_O_49_ continued to photocatalyse the decomposition of Rhodamine B at approximately twice the rate of Ta_3_N_5_.

In addition to evaluating the practical capabilities of the Ta_3_N_5_/W_18_O_49_ catalyst, the catalytic mechanisms responsible for dye oxidation were also investigated. By employing *tert*-butanol and *p*-benzoquinone as scavenger reagents, it was shown that whilst both hydroxyl and superoxide radicals contributed to cleavage of the Rhodamine B chromophore, the superoxide species alone were involved in N-deethylation of the dye molecules. Moreover, from the observed catalytic inactivity of W_18_O_49_ on its own and the marked influence of the scavenger reagents on decolourisation rate in the composite system, it was deduced that generation of both radical species at the W_18_O_49_ surface was permitted solely as a consequence of electronic transfer between W_18_O_49_ and Ta_3_N_5_ within the composite.

It is hoped that by building on the work presented herein, future studies will improve the Ta_3_N_5_/W_18_O_49_ composite still further by optimising the coverage of the Ta_3_N_5_ by nanostructured W_18_O_49_. In this way, and through scale-up of the synthesis techniques, it is the authors’ belief that Ta_3_N_5_/W_18_O_49_ composites hold significant promise as photocatalysts for dye degradation, and could provide an important contribution to the treatment of wastewater on a global scale.

## Methods

### Ta_3_N_5_ nanoparticles

A nanoparticulate powder of Ta_3_N_5_ was produced through thermal decomposition of TaCl_5_ under flow of ammonia gas at a rate of approximately 100 mL min^−1^. Guided by a protocol reported elsewhere^65^, TaCl_5_ (2.0 mmol), NaCl (2.8 mmol) and KCl (1.2 mmol) were ground to a fine powder using a mortar and pestle, then mixed inside a dry argon glove box by shaking together in a sealed glass vial. Once removed from the glove box, the mixed powder was quickly transferred to an alumina heating boat and heated at a rate of 10 °C min^−1^ to 800 °C inside a tube furnace, maintaining this temperature for ten hours under continuous flow of ammonia at a rate of approximately 100 mL min^−1^.

After cooling naturally to room temperature, the resultant red powder was reground and mixed with a further NaCl (11.2 mmol) and KCl (4.8 mmol), before being returned to the tube furnace for a second, identical 800 °C annealing step. This intermediate addition of NaCl and KCl flux was carried out to compensate for the loss of salt through evaporation, thereby ensuring that the forming Ta_3_N_5_ remained suspended in the molten salt mixture for the whole combined annealing time of 20 hours. The final product was centrifuged into deionised water six times to dissolve any residual NaCl, and then reground to a fine powder.

### Ta_3_N_5_/W_18_O_49_ nanocomposite

Nanowires of W_18_O_49_ were grown heterogeneously on **1** using a solvothermal approach. After grinding WCl_6_ into a fine powder inside a dry argon glove box, a solution of WCl_6_ (22.8 mmol dm^−3^) was prepared in a 4:1 volume mixture of ethanol and ethylene glycol (60 mL total volume); more specifically, the WCl_6_ powder was first dissolved in ethanol (5 mL) to generate a yellow solution, which was subsequently added to the remaining mixture of ethanol and ethylene glycol under rapid stirring. The ground powder of **1** (140 mg, 228 µmol) was added to this solution to obtain a 2:1 molar ratio of W to Ta in the reaction mixture, and the resulting red-orange suspension was stirred rapidly for several minutes. The suspension was transferred to a 125 mL PTFE cup which was in turn enclosed within a Parr acid digestion bomb and secured.

The sealed bomb was heated to 180 °C at a rate of 10 °C min^−1^, and was maintained at this temperature for 24 hours. After cooling naturally to room temperature, the flocculent blue precipitate was centrifuged in ethanol and then deionised water several times, before drying at 80 °C overnight. The brown solid obtained upon drying was collected and ground into a fine powder using a mortar and pestle.

### W_18_O_49_ control powder

A control powder of W_18_O_49_ was grown solvothermally as reported elsewhere^55^. An ethanolic solution of WCl_6_ (12.6 mmol dm^−3^, 70 mL) was prepared, transferred to a 125 mL PTFE cup and secured inside a Parr acid digestion bomb.

The sealed bomb was heated using the same temperature, heating rate and annealing time as in the preparation of **2**, and the resulting flocculent blue precipitate was centrifuged in ethanol and then deionised water, before drying at 80 °C overnight. The dried blue solid was collected and ground into a fine powder using a mortar and pestle.

### SEM and TEM characterisation

For SEM analysis, powders of **1** and **2** were deposited onto adhesive carbon tabs and examined using a Hitachi S4800 FE-SEM, operating at an accelerating voltage of 10 kV and an emission current of 10 µA.

Transmission electron microscopy (TEM) images were obtained using a TEM Jeol 2100 with a LaB_6_ source operating at an acceleration voltage of 200 kV. Micrographs were recorded on a Gatan Orius Charge-coupled device (CCD). The powders were sonicated in *n*-hexane (creating a turbid suspension) and drop-cast onto a 400 Cu mesh lacey carbon film grid (Agar Scientific Ltd) for TEM analysis.

### Identification of material phases

The crystal structures of **1** and **2** were investigated by transferring a small quantity of each powder into a 0.5 mm diameter glass capillary for measurement of the XRD diffractograms by a Bruker D8 Advance X-ray diffractometer. Diffractograms were measured using Cu-K_α_ radiation in conjunction with a Ni filter, employing steps of 0.015° at 10 s per step between 2*θ* angles of 10° and 70°.

### Band gap analysis using UV-Vis diffuse reflectance spectroscopy

For the determination of the optical band gaps of **1**, **2** and **3**, diffuse reflectance measurements of the powders were conducted using a spring-loaded powder cell loaded into an Agilent Cary 100 UV-Vis spectrophotometer, referencing the spectra to a Lapshere Spectralon diffuse reflectance standard. The spectra were recorded between wavelengths of 400 nm and 800 nm in the case of **1** and **2**, and 250 nm to 500 nm for **3** and an additional scan for **2**, using a step size of 1 nm and an integration time of 100 ms; spectra were measured using a visible quartz-iodide lamp for the samples of **1** and **2**, whereas for **3** and the second set of measurements for **2** a deuterium UV lamp was employed. Conversion of each diffuse reflectance spectrum to a Tauc plot was conducted using a Matlab program, and the band gap was calculated by extrapolating a line-of-best-fit through the point of steepest gradient within the absorption cut-off curve, as determined by the program.

### Chemical and electronic characterisation via XPS and UPS

To examine the stoichiometry of **1** and **2**, XPS spectra were recorded using a Kratos Axis Supra system utilising a monochromated Al K_α_ source. Each sample powder was compressed into a pellet using a custom-built press and mounted on an adhesive carbon tab, thereby preventing the underlying substrate from contributing to the spectra. Survey spectra were measured over binding energies in the range 0–1200 eV using pass energy 160 eV, dwell time 100 ms and step size 1 eV. Core level peaks of interest were measured more precisely using pass energy 20 eV, dwell time 250 ms and step size 100 meV, and the spectra were averaged over up to five scans until a signal-to-noise ratio of 100 or greater was attained.

For each XPS core peak spectrum, a Shirley function was assumed for the secondary electron background and peaks were fitted using Gaussian-Lorentzian components. Within a given peak, the full widths at half maximum of the constituent fitting components were set equal. In the case of a doublet, spin population statistics were considered to set the requisite relative area ratios of the two states within each pair of doublet fitting components. The atomic ratios of different elements were compared by dividing the area of each peak by its corresponding relative sensitivity factor.

The position of the valence band edges of **1**, **2** and **3** relative to the Fermi level were investigated by performing further XPS scans at binding energies close to 0 eV; these measurements again employed a pass energy of 20 eV, but the step size was reduced to 50 meV, dwell time was increased to 3 s, and the counts were averaged over five scans, regardless of the signal-to-noise ratio. To ensure electrical contact between the samples and the metallic sample holder, copper clips were screwed onto the holder in contact with the powder and the top of the carbon tab.

To compensate for differential surface charging, an electronic charge neutraliser was employed using a charge balance potential of 3.3 V, filament bias 1.0 V and filament current 0.4 A. The spectra were subsequently “carbon-corrected”: after referencing the C1s peak to its generally-accepted binding energy value of 284.8 eV, the same shift was applied to the binding energies of all associated spectra (spectra corresponding to the same sample location) to account for the surface potential resulting from charge compensation.

UPS measurements were performed using the same carbon tab-mounted powder samples as for the XPS valence band measurements, again with copper clips to provide electrical contact to the instrument. Surface contaminants were removed by bombarding a 2 mm × 2 mm square with 1000+ atom argon clusters of energy 10 keV for 60 s. Within the subsequent etch-crater, measurements over electron kinetic energies in the range 0.4–26.0 eV were undertaken using a He I source (21.22 eV photon energy), with 5 eV pass energy, 50 meV step size, 60 ms dwell time and a microscope aperture of diameter of 55 µm, while additional measurements between kinetic energies of 10.0 eV and 26.0 eV were performed using the same source, pass energy and step size, but 200 ms dwell time and an aperture of diameter 110 µm. All measurements employed a −9 V sample bias potential.

### Dye degradation experiments

The photocatalytic properties of **1**, **2** and **3** were gauged by suspending the powder (5 mg) in an aqueous solution of Rhodamine B (0.02 mM, 10 mL) and measuring the absorption by the solution over UV and visible wavelengths following different durations of stirring under white light illumination. A control solution of Rhodamine B (0.02 mM, 10 mL) was also prepared which contained no suspended catalyst. The white light illumination was provided by a custom-built mirror-walled light box containing ten white light fluorescent tubes, each of power 18 W and a colour temperature rating of 6500 K, as well as a fan to provide cooling^31, 33^.

Prior to illumination, each suspension was stirred rapidly for one hour in darkness to allow for adsorption of the dye. After each illumination step, the suspensions were centrifuged to precipitate the solid component, and an aliquot of the supernatant was transferred to a 2.5 mL plastic cuvette and loaded into an Agilent Cary 100 UV-Vis spectrophotometer. Using a cuvette containing de-ionised water as a reference, the absorption by each dye sample was measured over wavelengths in the range 400–650 nm, with a step size of 1 nm and integration time 100 ms. The solution was subsequently returned to its corresponding suspension. For each absorption spectrum, a baseline was subtracted from the measurements by using a Matlab program to plot a straight line tangentially between points close to the absorption minimum on either side of the absorption peak.

To assess the cyclability of each catalyst, the suspensions were exposed to white light for an additional 17 hours (yielding a total illumination time of 22.5 hours) before being centrifuged several times into deionised water then re-suspended in a fresh aqueous Rhodamine B solution (0.02 mM, 10 mL). Decolourisation of the dye was subsequently investigated using the same sequence of illumination and absorption testing as for the as-synthesised catalysts.

The mechanisms of photocatalysis were investigated through use of scavenger reagents. A sequence of stepwise illumination and UV-Vis absorption measurements was carried out as previously for suspensions of **1** and **2** (10 mg) in Rhodamine B solution (0.02 mM, 10 mL), containing either no scavenger reagent, *p*-benzoquinone (1 mM) or *tert*-butanol (10 mM).

## Electronic supplementary material

Sup info
