# Supplementary material for: Active removal of waste dye pollutants using Ta3N5/W18O49 nanocomposite fibres
Source: Sci Rep. 2017 Jun 22;7:4090. doi: 10.1038/s41598-017-04240-4 (PMC5481444; doi:10.1038/s41598-017-04240-4)
Supplement: Supplementary file 1 — Sup info [file 41598_2017_4240_MOESM1_ESM.pdf]

Electronic Supplementary Information

For

Active removal of waste dye pollutants using $Ta_3N_5/W_{18}O_{49}$ nanocomposite fibres

Daniel R. Jones,[1] Virginia Gomez,[1] Joseph C. Bear,[2] Bertrand Rome,[1] Francesco Mazzali,[3] James D. McGettrick,[4] Aled R. Lewis,[5] Serena Margadonna,[3] Waheed A. Al-Masry[6] and Charles W. Dunnill[1*]

[1] Energy Safety Research Institute (ESRI), Swansea University Bay Campus, Swansea SA1 8EN, UK.
[2] Materials Chemistry Centre, Department of Chemistry, University College London, 20 Gordon Street, London, WC1H 0AJ, UK.
[3] College of Engineering, Swansea University Bay Campus, Swansea SA1 8EN, UK.
[4] SPECIFIC, Swansea University Bay Campus, Swansea SA1 8EN, UK.
[5] Systems and Processing Engineering Centre (SPEC), Swansea University Bay Campus, Swansea SA1 8EN, UK.
[6] Department of Chemical Engineering, King Saud University, Riyadh, Saudi Arabia.
[*] Email: C.Ddunnill@Swansea.ac.uk

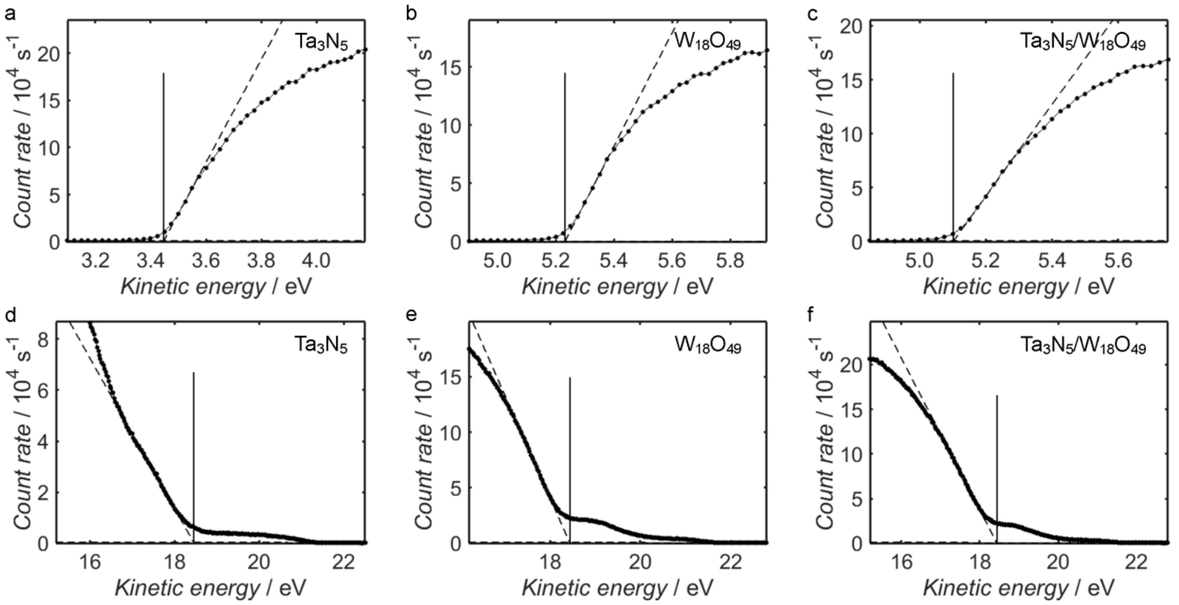

Fig. S1 Ultraviolet photoelectron spectroscopy (UPS) measurements showing the fitted secondary electron onsets (**a**-**c**) and valence band edges (**d**-**f**) of the spectra depicted in Fig. 5**d**-**f** of the paper. The measurements for $W_{18}O_{49}$ (**b** and **e**) and the $Ta_3N_5/W_{18}O_{49}$ composite (**c** and **f**) yielded similar extrapolated values of E_{SEO} and E_V, thereby resulting in comparable estimates for E_{IP} and ϕ; this similarity is indicative of the core-shell structure of the composite, with the Ta_3N_5 component present as the core material within a $W_{18}O_{49}$ shell. The ϕ estimate for Ta_3N_5 obtained from **a** was found to be inconsistent with the corresponding E_{IP} value and the estimated difference E_F-E_V from Fig. 5**a** of the paper; this disparity was attributed to the poor conductivity of Ta_3N_5 leading to surface charging of the material, invalidating the assumption of a constant Fermi level between instrument and sample.

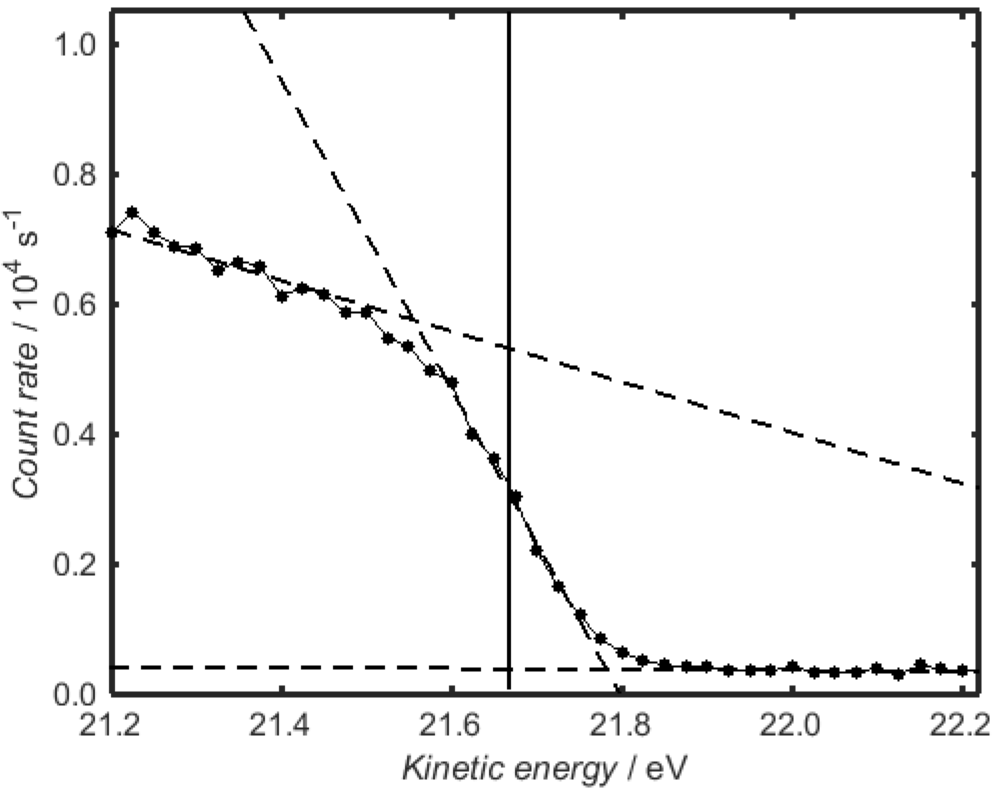

Fig. S2 UPS measurements close to the Fermi energy of the metallic sample holder, which was in electrical contact with the surface of each sample. The Fermi energy, corresponding to the solid vertical line, is defined as the point at which states have a 50 % probability of occupation, so may be estimated as the energy halfway between the points at which a linear fit through the Fermi edge intersects a linear fit through the points of 100 % occupation at lower kinetic energy and another linear fit through the baseline.

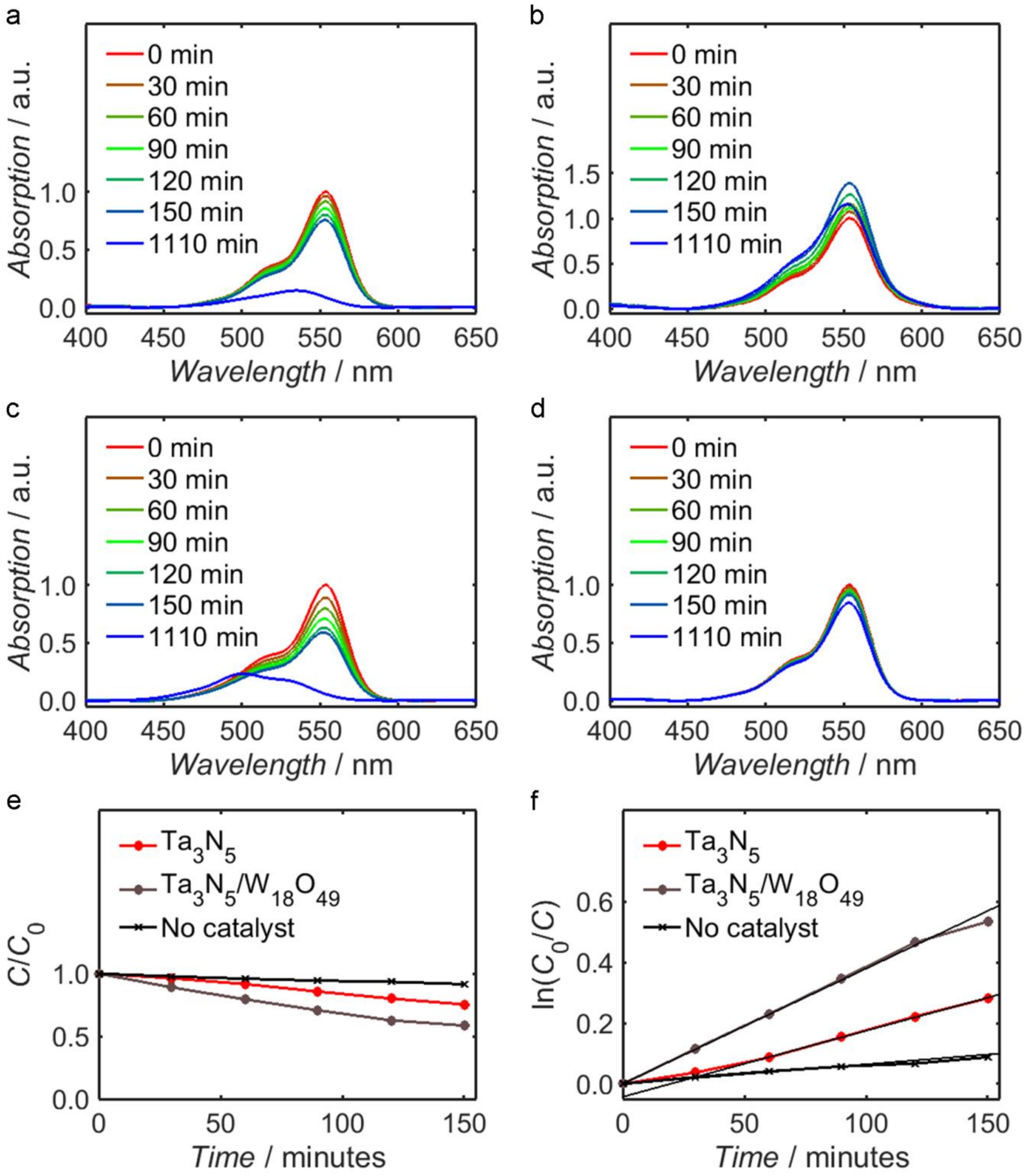

Fig. S3 UV-Vis absorption spectra of supernatant extracted from white light illuminated suspensions of recycled Ta_3N_5 (**a**) , $W_{18}O_{49}$ (**b**) and $Ta_3N_5/W_{18}O_{49}$ (**c**) in Rhodamine B solution (0.02 mM), and a control solution containing no catalyst (**d**). The catalysts were recycled from the suspensions corresponding to Fig. 8 of the paper through repeated centrifugation into deionised water. As in the case prior to recycling, $W_{18}O_{49}$ demonstrated negligible catalytic behaviour towards Rhodamine B. The relationships between C/C_0 and illumination time depicted in **e** show that in addition to producing the most pronounced rate of N-deethylation, recycled $Ta_3N_5/W_{18}O_{49}$ again exhibited the highest rate of overall decolourisation. By plotting C_0/C logarithmically as a function of illumination time in **f**, the rate constant for oxidation of the dye by $Ta_3N_5/W_{18}O_{49}$ is estimated to be approximately twice the corresponding estimate for Ta_3N_5.

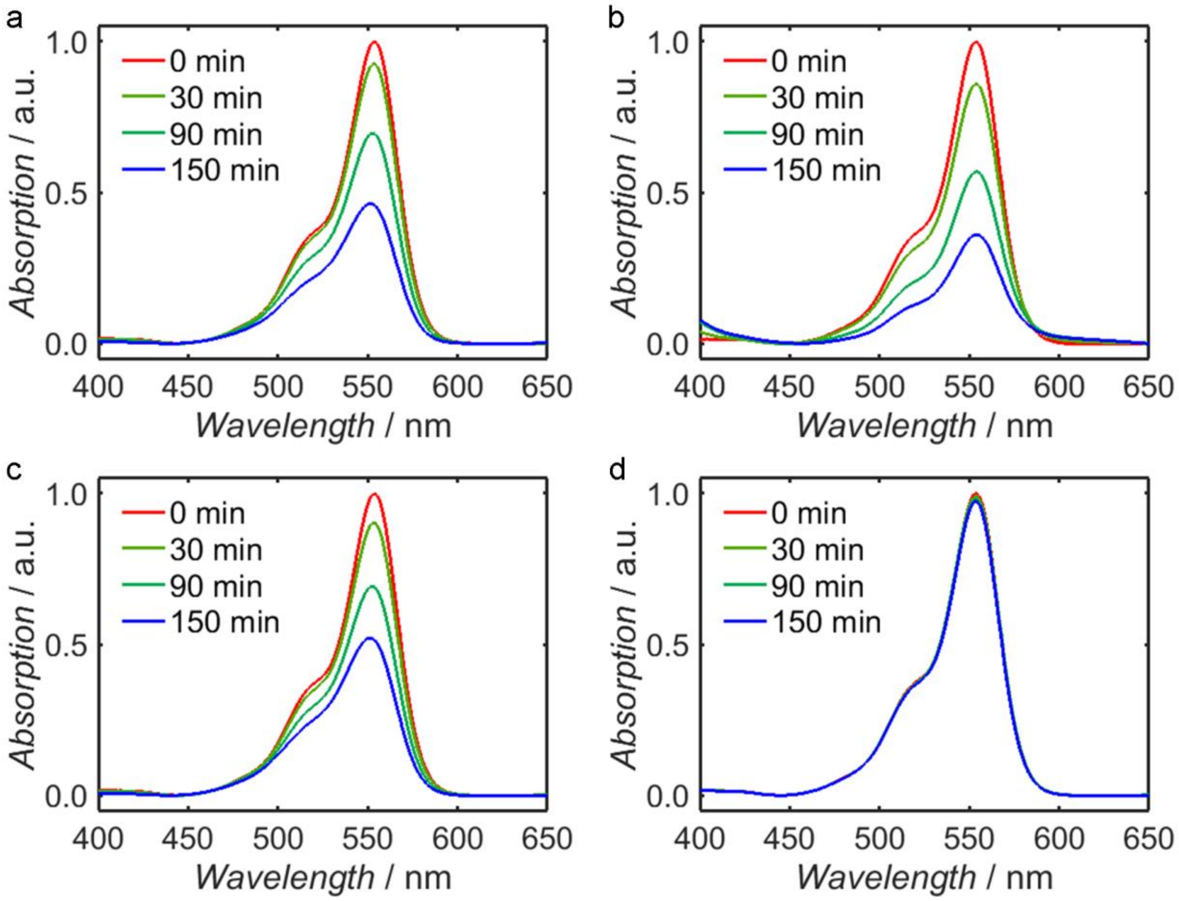

Fig. S4 UV-Vis absorption spectra of supernatant from suspensions of Ta_3N_5 in Rhodamine B solutions (0.02 mM) containing no scavenger reagent (**a**), or either *p*-benzoquinone (1 mM) (**b**) or *tert*-butanol (10 mM) (**c**) as scavengers of superoxide or hydroxyl radicals, respectively. The aliquots of supernatant were extracted after different periods of white light illumination, and a control solution containing no catalyst or scavenger reagents was also investigated (**d**). The most rapid decolourisation resulted from the presence of *p*-benzoquinone, while the absence of significant hypsochromic shifts of the absorption peaks in **a**-**c** suggests that negligible N-deethylation occurred in all three sample suspensions.

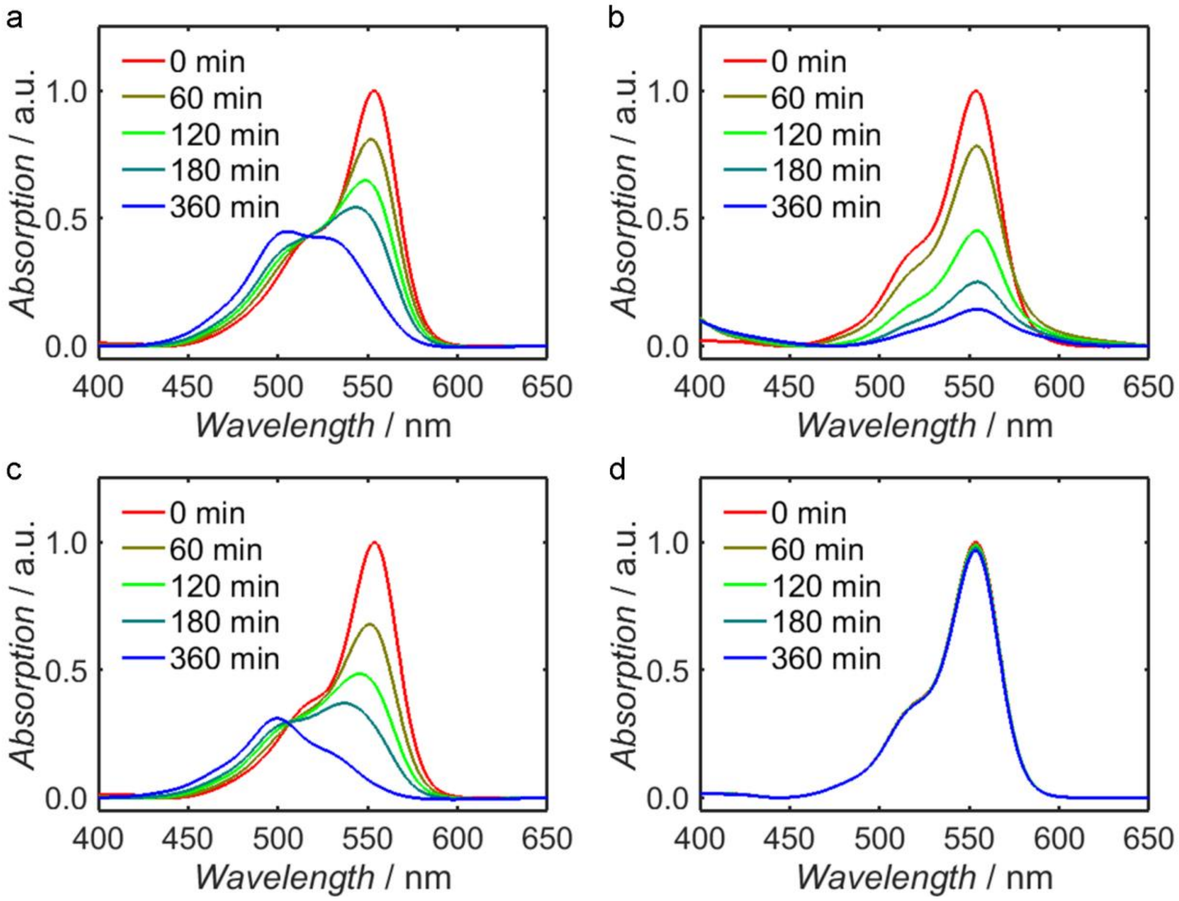

Fig. S5 UV-Vis absorption spectra of supernatant from suspensions of $Ta_3N_5/W_{18}O_{49}$ in Rhodamine B solutions (0.02 mM) containing no scavenger reagent (**a**), or either *p*-benzoquinone (1 mM) (**b**) or *tert*-butanol (10 mM) (**c**) as scavengers of superoxide or hydroxyl radicals, respectively. The aliquots of supernatant were extracted after different periods of white light illumination, and a control solution containing no catalyst or scavenger reagents was also investigated (**d**). The introduction of *p*-benzoquinone suppressed N-deethylation while also increasing the rate of decolourisation, whereas the system containing *tert*-butanol exhibited enhanced decolourisation but also a hypsochromic shift indicative of N-deethylation.

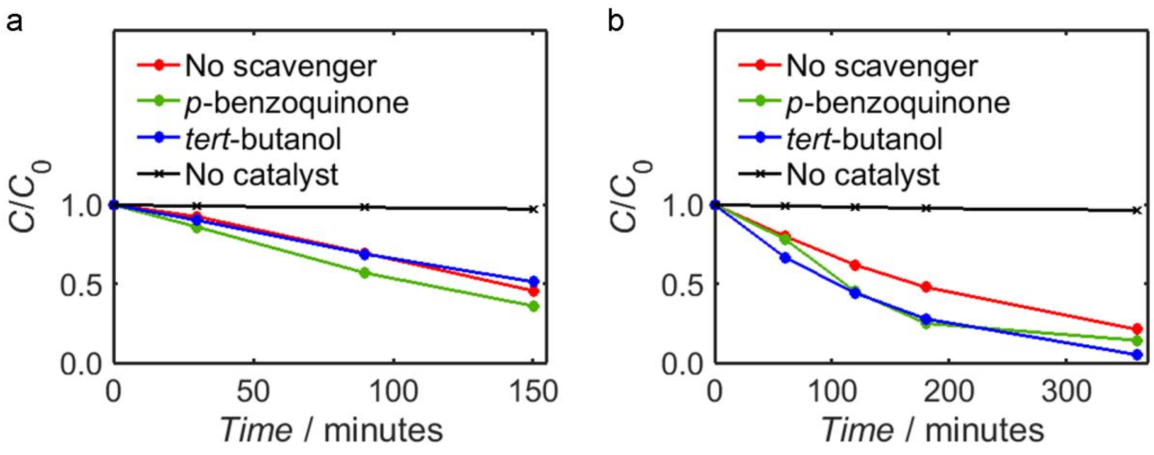

Fig. S6 The variation of C/C_0 as a function of illumination time for the supernatant of suspensions containing Ta_3N_5 (**a**) or $Ta_3N_5/W_{18}O_{49}$ (**b**), Rhodamine B (0.02 mM) and either no scavenger reagent, *p*-benzoquinone (1 mM) or *tert*-butanol (10 mM). Only *p*-benzoquinone had a significant effect on the rate of decolourisation in the case of Ta_3N_5, whereas both scavenger reagents produced more rapid decolourisation in suspensions containing $Ta_3N_5/W_{18}O_{49}$.
